# Impaired development of the cerebral cortex in infants with congenital heart disease is correlated to reduced cerebral oxygen delivery

**DOI:** 10.1038/s41598-017-14939-z

**Published:** 2017-11-08

**Authors:** Christopher J. Kelly, Antonios Makropoulos, Lucilio Cordero-Grande, Jana Hutter, Anthony Price, Emer Hughes, Maria Murgasova, Rui Pedro A. G. Teixeira, Johannes K. Steinweg, Sagar Kulkarni, Loay Rahman, Hui Zhang, Daniel C. Alexander, Kuberan Pushparajah, Daniel Rueckert, Joseph V. Hajnal, John Simpson, A. David Edwards, Mary A. Rutherford, Serena J. Counsell

**Affiliations:** 1 Centre for the Developing Brain, Division of Imaging Sciences and Biomedical Engineering, King’s College London, London, United Kingdom; 2 Biomedical Image Analysis Group, Department of Computing, Imperial College London, London, United Kingdom; 3 Paediatric Cardiology Department, Evelina London Children’s Hospital, St Thomas’ Hospital, London, United Kingdom; 4 Department of Computer Science and Centre for Medical Image Computing, University College London, London, United Kingdom; 5 Division of Imaging Sciences and Biomedical Engineering, King’s College London, London, United Kingdom

## Abstract

Neurodevelopmental impairment is the most common comorbidity associated with complex congenital heart disease (CHD), while the underlying biological mechanism remains unclear. We hypothesised that impaired cerebral oxygen delivery in infants with CHD is a cause of impaired cortical development, and predicted that cardiac lesions most associated with reduced cerebral oxygen delivery would demonstrate the greatest impairment of cortical development. We compared 30 newborns with complex CHD prior to surgery and 30 age-matched healthy controls using brain MRI. The cortex was assessed using high resolution, motion-corrected T2-weighted images in natural sleep, analysed using an automated pipeline. Cerebral oxygen delivery was calculated using phase contrast angiography and pre-ductal pulse oximetry, while regional cerebral oxygen saturation was estimated using near-infrared spectroscopy. We found that impaired cortical grey matter volume and gyrification index in newborns with complex CHD was linearly related to reduced cerebral oxygen delivery, and that cardiac lesions associated with the lowest cerebral oxygen delivery were associated with the greatest impairment of cortical development. These findings suggest that strategies to improve cerebral oxygen delivery may help reduce brain dysmaturation in newborns with CHD, and may be most relevant for children with CHD whose cardiac defects remain unrepaired for prolonged periods after birth.

## Introduction

Congenital heart disease (CHD) is the most common congenital disorder in newborns, affecting approximately 1% of births^1^. Of these, approximately half will have severe or moderately severe forms of CHD that require expert cardiology care at birth^2^. Survival rates through adolescence have improved dramatically^3^, and neurodevelopmental impairment has now become the most important comorbidity in this growing population of survivors. Up to half of children with complex CHD experience a distinct pattern of neurodevelopmental and behavioural impairment, characterised by mild cognitive impairment, impaired social and communication skills, inattention, impulsive behaviour and later, impaired executive function^4^.

Understanding of the biological mechanisms underlying neurodevelopmental delay in this population remains limited. Early animal catheterisation studies suggested that fetal cerebral oxygen delivery is reduced in CHD, particularly in transposition of the great arteries (TGA) and left-sided lesions^5^. Advances in fetal MRI have since demonstrated a 10% reduction in ascending aortic saturations in a mixed group of CHD^6^, which may impair normal oligodendrocyte maturation in the developing brain^7^.

Brain “immaturity” has been described in CHD before surgery^8^ using a radiologist-graded scoring system^9^, and has been associated with impaired neurodevelopment at 2 years of age in children with CHD^10^. An objective marker of the developing brain’s cortical folding is the gyrification index, first described in the context of autopsy specimens^11,12^, and has been shown to increase markedly over the third trimester and during early infancy^13^. Gyrification index may be an important metric when studying neurodevelopment in this population: reduced gyrification index of newborns with CHD has been demonstrated in autopsy specimens^14^, fetal MR imaging studies^15^, and neonatal MR imaging studies^16,17^, although all studies to date have required varying degrees of manual input, and neonatal studies to date have not attempted to quantify regional differences or to correlate cortical development with cerebral oxygenation delivery.

We hypothesised that impaired cerebral oxygen delivery (CDO_2_) in infants with CHD is a cause of impaired cortical development, and predicted that cardiac lesions most associated with most reduced cerebral oxygen delivery would demonstrate the greatest impairment of cortical development. We aimed to compare the cortex of newborns with CHD prior to surgery versus healthy control infants, assessing cortical folding, cortical grey matter volumes and cerebral blood flow calculated from high resolution magnetic resonance imaging (MRI).

## Results

### Demographics

There were no significant differences in GA at birth or at scan between newborns with CHD and healthy controls. There were no significant differences in birthweight, birthweight z-score, head circumference at birth, head circumference z-score, and sex between groups. There were no sex-based differences in total grey matter volume, gyrification index, cerebral oxygen delivery or regional cerebral oxygen saturations. A summary of CHD diagnoses can be seen in Table 1.

**Table 1:** Cohort characteristics.

Variable	Control Newborns n = 30	Newborns with Congenital Heart Disease n = 30	p value
Gestational age at birth (weeks)	38.9 (38.1–39.3)	38.4 (37.9–38.9)	0.07
Post-menstrual age at scan (weeks)	39.1 (38.7–39.7)	39.3 (38.7–39.6)	0.81
Male	16	8	0.06
Birth weight (g)	3220 (2920–3550)	3125 (2800–3500)	0.37
Birth weight z-score	−0.02 (−0.39–0.5)	−0.17 (−0.77–0.49)	0.69
Birth head circumference (cm)	34.5 (33–35.5)	34 (33–35)	0.24
Birth head circumference z-score	0.72 (−0.06–1.20)	0.05 (−0.49–0.76)	0.11
Heart lesion – no. (%)			
- Transposition of the great arteries (TGA)	—	14 (47)	
- TGA requiring septostomy (% TGA)		5 (36)	
- Coarctation of the aorta	—	4 (13)	
- Hypoplastic left heart syndrome	—	3 (10)	
- Pulmonary atresia	—	3 (10)	
- Tetralogy of Fallot	—	3 (10)	
- Pulmonary stenosis	—	2 (7)	
- Truncus arteriosus	—	1 (3)	

GA at birth and PMA at scan are presented as median (interquartile range). Apgar scores reflect condition at birth, ranging from 0 to 10, with lower scores indicating a worse clinical condition. p values calculated using Mann–Whitney U test for continuous data, and Fisher’s exact test for categorical variables. z-scores for head circumference and birth weight calculated using UK-WHO 2006 reference data.

### Cerebral oxygen delivery (CDO_2_) is positively associated with brain volume and gyrification

**Figure 1:**
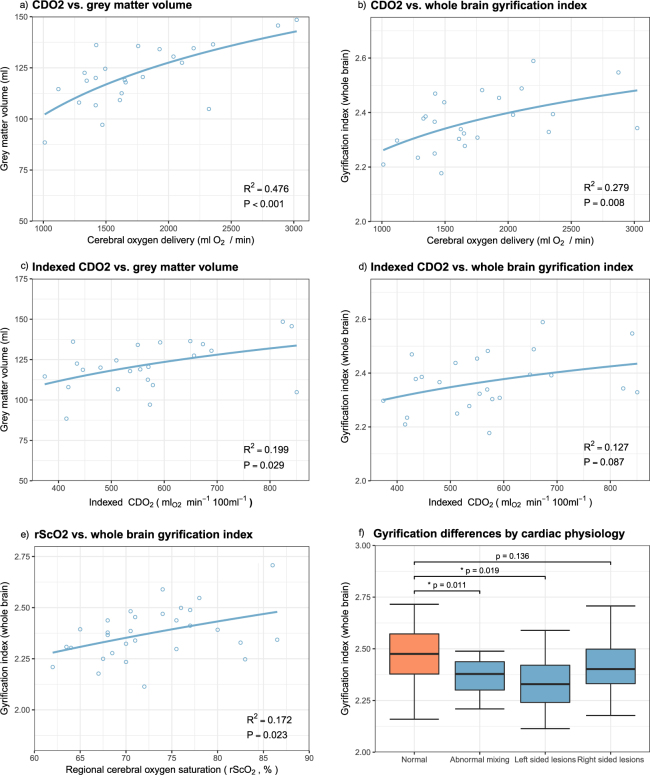
Cerebral oxygen delivery (CDO2) demonstrates a positive association with grey matter volume (**a**) and whole brain gyrification (**b**). These trends persist after indexing CDO2 per 100 ml brain volume (**c** and **d**). Regional cerebral oxygen saturation has a limited positive relationship with gyrification index (**e**). Abnormal mixing lesions and left sided lesions demonstrate a significantly lower gyrification index, while right-sided lesions are less affected (**f**).

Phase contrast measurements, preductal arterial saturations and haemoglobin levels were successfully obtained in 24 of 30 babies with CHD (mean 161, SD 21 g/L). Calculated CDO_2_ (median 1638 ml O_2_/min, range 1011–3023 ml O_2_/min) showed a positive association with total brain volume (R^2^ = 0.42, p < 0.001), grey matter volume (R^2^ = 0.48, p < 0.001, Fig. 1a), and whole brain gyrification index (R^2^ = 0.279, p = 0.008, Fig. 1b). A secondary analysis included days of mechanical ventilation and requirement for prostaglandin E2 in the regression model with no significant effect on the results for brain volume, grey matter volume or gyrification. Indexing CDO2 per unit of brain volume retained the same trends as without indexing, but weakened both the association with grey matter volume (R^2^ = 0.199, p = 0.029, Fig. 1c) and with gyrification index (R^2^ = 0.127, p = 0.087, Fig. 1d).

To understand the relative contribution of CBF and oxygen saturations to CDO2, we compared correlations between both factors and CDO2 (Supplementary Figure S1). CBF was more strongly correlated with CDO2 (R^2^ = 0.643, p < 0.001) than arterial saturations (R^2^ = 0.107, p = 0.119), suggesting that blood flow contributed more to the values seen for CDO2. Subgroup analysis of cerebral haemodynamics by lesion physiology was hampered by small sample sizes, although there was a trend towards higher cerebral blood flows and oxygen delivery in right sided lesions and lower in CHD with abnormal mixing (Supplementary Figure S2).

Regional cerebral oxygen saturation (rScO_2_) was measured in all 30 babies with CHD. Consecutive repeat measurements were obtained in 24 of the 30 babies, demonstrating good repeatability (linear R^2^ = 0.86, p < 0.001). rScO_2_ showed a modest correlation with both whole brain gyrification index (R^2^ = 0.17, p = 0.023, Fig. 1e), and grey matter volume (R^2^ = 0.21, p = 0.011).

### Cortical volumes are reduced in newborns with CHD

Infants born with CHD had smaller total brain volumes (p < 0.001) and smaller cortical grey matter volumes (p < 0.01). Other brain volumes were also smaller, including deep grey matter (p < 0.001), white matter (p = 0.01), and cerebellar volume (p < 0.001). Ventricular volume was not significantly different between groups (p = 0.09). Extracerebral CSF space was increased in the CHD group (p = 0.011). Volumetric results are summarised in Table 2.

**Table 2:** Volume differences between newborns with congenital heart disease and healthy controls.

Region	Volume (ml), mean (SD)		ANCOVA	
	CHD, n = 30	Control, n = 30	p value	
Whole brain	308 (29.3)	335 (33.9)	<0.001	*
Cortical grey matter	122 (14.4)	132 (17.4)	0.003	*
Frontal grey matter	41 (5.0)	44 (5.6)	0.011	*
Parietal grey matter	28 (3.2)	31 (4.5)	0.002	*
Temporal grey matter	25 (3.3)	28 (3.9)	<0.001	*
Occipital grey matter	19 (2.7)	21 (2.8)	0.014	*
Cerebellum	20 (2.3)	22 (2.5)	0.073	Ns
Extra-axial CSF space	78 (20.8)	68 (10.8)	0.011	*

Comparison of groups performed with multivariate general linear models, with PMA included as a covariate. Exploratory regional analyses displayed with *significance and Ns = not significant. Significance did not change with the addition of weight at scan as a covariate.

### Gyrification index is globally reduced in newborns with CHD

**Figure 2:**
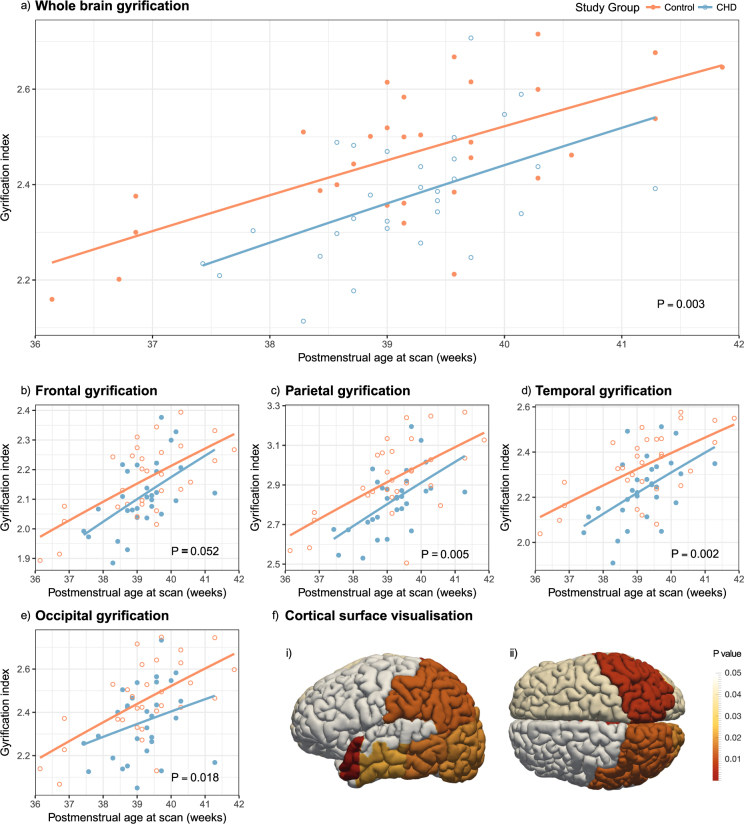
Gyrification index differences between newborns with complex congenital heart disease (open blue) and healthy controls (solid orange), in the whole brain (**a**) and exploratory regional analysis (**b**–**e**). The cortical surface visualisation (**f**) demonstrates regions where gyrification is reduced in newborns with congenital heart disease compared to healthy term controls, from the left lateral side (i) and from above (ii); colours represent p values from multivariate general linear models, using postmenstrual age as a covariate; no multiple comparisons correction has been performed in this visualisation.

Brain gyrification was reduced in infants with CHD (p < 0.01, Fig. 2a). Regional analysis showed significantly reduced gyrification index in the temporal (p = 0.002), parietal (p = 0.005) and occipital (p = 0.018), and a trend towards reduction in the frontal lobes (p = 0.052). Regional gyrification differences between groups are described in Table 3, with plots displayed in Fig. 2b–e and a visualisation on a representative cortical surface in Fig. 2f.

**Table 3:** Differences in gyrification index between newborns with congenital heart disease and healthy controls.

Region	GI mean (SD)		ANCOVA	
	CHD, n = 30	Control, n = 30	p value	
Whole brain	2.373 (0.127)	2.464 (0.144)	0.003	*
Frontal lobes	2.112 (0.114)	2.164 (0.124)	0.052	Ns
Parietal lobes	2.820 (0.155)	2.930 (0.194)	0.005	*
Temporal lobes	2.233 (0.145)	2.338 (0.151)	0.002	*
Occipital lobes	2.355 (0.167)	2.454 (0.179)	0.018	Ns

Comparison of groups performed with multivariate general linear models, with PMA included as a covariate. For regional comparisons, p = 0.0125 used as a Bonferroni correction threshold, with *representing significance and Ns = not significant.

### Gyrification varies between different CHD types

To explore differences further, newborns with CHD were divided into three main physiological groups: 1) Abnormal mixing (i.e. transposition of the great arteries, double-outlet right ventricle), 2) Left sided lesions (i.e. hypoplastic left heart syndrome, coarctation of the aorta), and 3) Right sided lesions (i.e. pulmonary atresia or stenosis), and each group compared to its matched controls. Compared to healthy newborns, gyrification was reduced in mixing (p = 0.011) and left sided lesions (p = 0.019), but not in right sided lesions (p = 0.136) (Fig. 1f).

### Requirement for septostomy is not associated with cortical volume and gyrification differences

Infants with TGA required septostomy in 5 of 14 cases (36%). We repeated analyses with this as a co-variate in the statistical analysis and found results were unchanged. Within the TGA group, we compared infants with and without a requirement for septostomy, and found no difference in terms of brain volume (p = 0.825) or gyrification (p = 0.19).

## Discussion

Reduced cerebral oxygen delivery in newborns with CHD before surgery is associated with impaired cortical development. Cortical grey matter volumes and gyrification index were lower in newborns with complex CHD when compared to healthy matched controls. The degree of cortical impairment was most significant in mixing and left-sided lesions, which may relate to the fetal cerebral circulatory impairment that has been demonstrated in these groups^5,6^.

A recent study of a similar number of infants showed reduced CDO_2_ in newborns with mixed complex CHD compared to controls, with the lowest CDO_2_ measured in infants with transposition^18^. Lim and colleagues found that arterial saturations had the greatest influence on CDO2, and that there were no significant CBF differences between CHD and control infants^18^. We were unable to replicate these findings due to lack of flow measurements in our control group, but our results suggest that CBF had a greater contribution to the variation of CDO2 within our CHD cohort. Ascending aorta oxygen saturations were found to be 10% lower in fetuses with CHD when compared to healthy controls using fetal MRI^6^. Animal studies have shown that a lower oxygen tension environment affects mechanisms that coordinate myelination and angiogenesis during the early phase of brain development^7^, and causes diminished proliferation and neurogenesis in the subventricular zone, accompanied by reduced cortical growth^19^. Taken together with our results, these findings suggest that the developing brain may be adversely affected by the lower oxygen tension environment that is observed in CHD in both fetal and postnatal life.

A limited number of studies have reported altered cortical folding in fetuses and newborns with CHD. An “immature cortical mantle” was first described in an early post-mortem study from a cohort of 41 infants with hypoplastic left heart syndrome^14^. Delayed cortical folding has been described using a radiologist scoring system^9^ in pre-surgical newborns with CHD, starting at around 30 weeks of gestation^8^. The same finding in post-surgical infants has been shown to be a strong predictor of later neurodevelopmental outcome^10^. Specific analysis of the gyrification of the opercula was performed in a cohort of newborns with HLHS and TGA prior to surgery, and demonstrated that opercula in CHD was more “open” and exhibited reduced folding complexity^20^. Gyrification index in fetuses with HLHS was found to be reduced compared to controls using a semi-automated analysis technique, and these group differences increased progressively with advancing gestational age^15^. Our study adds weight to these findings in a more diverse group of CHD.

Cortical development follows a predictable timeline^13^. Most gyri become well defined between 26–28 weeks, becoming more prominent and deeply infolded during the third trimester, with subsequent development of secondary and tertiary gyri^21^. The frontal third of the brain commences its gyrification slightly before the intermediate and caudal thirds, but also takes longer to reach its adult degree of cortical folding by 48 weeks (compared to 44 and 43 weeks respectively)^13^. We found that gyrification index in our CHD group was most reduced in parietal and temporal regions, and least in the frontal region. This may be due to later completion of gyrification in the frontal region, but also that the pace of gyrification is greatest in parietal, temporal and occipital regions during the time window of our study^13^. Impaired gyrification in infants with CHD may therefore be most apparent in these regions.

To understand gyrification differences further, we examined three subgroups using a cardiac physiology-based categorisation: abnormal mixing, left sided lesions. and right sided lesions. We found significant gyrification differences in the abnormal mixing group, but were unable to form strong conclusions from our left- and right-sided lesion groups due to small subgroup numbers. We were unable to study infants with hypoplastic left heart syndrome separately, a condition known to impair fetal brain development^15^. There are limited previous studies for comparison, with no pre-surgical neonatal studies for reference. In a previous study of post-surgical infants with transposition, gyrification was reported to be similar to controls^17^, which may be explained by smaller group sizes and less closely matched postmenstrual ages at scan. There was unfortunately no control group for their pre-surgery infants for direct comparison.

Our finding of reduced brain volumes in newborns with transposition fits with a recent large population study of 924,422 Dutch liveborn singletons, which found that in contrast to other forms of CHD where both head circumference and birth weight of infants were reduced, only infants with transposition had smaller head circumference relative to birth weight^22^. Reduced head circumference at birth, the most widely available proxy of impaired fetal brain growth^23^, has often been reported in CHD, most consistently in HLHS and TGA^24–27^, but also in tetralogy of Fallot, ventricular septal defects, common arterial trunk, and anomalous pulmonary venous return^22^. In contrast to other studies, we found that there was no difference in the z-scores of head circumferences between CHD and healthy groups in our cohort. However, brain volumes were significantly reduced in CHD. The discrepancy between head circumference and brain volume is explained by a larger extra-cerebral CSF volume in the CHD group, in the presence of comparable ventricular volumes.

Brain growth trajectories in CHD have been shown to diverge from healthy fetuses in the third trimester, during a period where there is usually an acceleration of energy-demanding brain growth^28^, using both fetal ultrasound^29^ and fetal MRI^6,15,28^. A limited number of cohorts have also suggested an increased prevalence of ventriculomegaly in fetuses with CHD, as assessed by radiologist assessment^30^ and two-dimensional measurements^31^. Increased CSF spaces in CHD has only previously been described in a limited number of studies^28,31^, and may be a marker of cerebral parenchymal growth disturbance.

Our study has a number of limitations. We did not have phase contrast measurements for our control group, limiting analysis of CDO2 against cortical metrics to those infants with CHD. In addition, our CDO2 measurements were performed shortly following birth, while the majority of brain development until that point occurs in utero with a fetal circulation. Delayed brain growth and maturation is more like to reflect CDO2 in utero, which was not measured in this study. There are many influences on early brain growth, and although currently poorly understood, genetic abnormalities are highly prevalent in the CHD population^32^. It is logical that smaller brains as a direct result of genetic factors with lower metabolic demands will require less blood flow and oxygen delivery, which may explain part of the association demonstrated in this study. We addressed this by calculating the indexed oxygen delivery per unit of brain tissue, and our results support the contention that CDO2 is reduced even when taking into account differences in brain volume. However, it is not possible to fully determine if lower CDO2 as a result of CHD has resulted in the development of a smaller brain, or if extrinsic factors to this analysis (i.e. genetic) have resulted in smaller brains with smaller metabolic demands that require less CDO2.

## Conclusions

Cortical folding and cortical grey matter volume is reduced in newborns with congenital heart disease when compared to healthy matched controls. Lower cerebral oxygen delivery measured in newborns with CHD prior to surgery is associated with reduced cortical grey matter volume and gyrification. This supports the possibility that strategies to improve cerebral oxygen delivery in infants with CHD could modify the derailing trajectory of brain development. Our finding of reduced CDO2 may be of greatest importance for children with CHD whose heart defects remain unrepaired for long periods of time after birth, exacerbating deficits in oxygen and other metabolic substrate supply that may have occurred during the prenatal period, and leading to further decrements in brain growth and development after birth.

## Methods

The project was approved by the National Research Ethics Service West London committee (CHD: 07/H0707/105, Controls: 14/LO/1169) and informed written parental consent was obtained prior to imaging. All methods and experiments were performed in accordance with relevant guidelines and regulations.

### Participants

A prospective cohort of 33 infants born with complex CHD requiring surgery within one year was recruited from the Neonatal Intensive Care Unit at St Thomas’ Hospital, London. Two infants were found to have neonatal arterial ischaemic stroke on MRI (left parietal stroke (n = 1) and left frontal stroke (n = 1); both TGA post-septostomy) and were excluded from this analysis. A further infant (TGA) was excluded due to unknown date of last menstrual period and lack of ultrasound dating scan.

We therefore studied 30 infants with CHD, born at a median (range) gestational age (GA) of 38^+3^ (34^+6^–40^+4^) weeks. A group of healthy controls was matched by GA at birth and at scan, contemporaneously recruited from the postnatal ward at St Thomas’ Hospital through the Developing Human Connectome Project^33^, born at a median (range) GA of 38^+6^ (35^+2^–40^+6^) weeks. The median GA at imaging was 39^+2^ (37^+3^–41^+4^) weeks for the CHD group and 39^+1^ (36^+1^–41^+6^) weeks for the control group.

### MR imaging

T2-weighted, T1-weighted and phase contrast angiography MR imaging was performed on a Philips Achieva 3 Tesla system (Best, The Netherlands) with a 32-channel neonatal head coil and neonatal positioning device^33^, situated on the neonatal intensive care unit at St Thomas’ Hospital, London. All examinations were supervised by a paediatrician experienced in MR imaging procedures. All infants were scanned in natural sleep without sedation. Pulse oximetry, respiratory rate, temperature and electrocardiography were monitored throughout. Ear protection comprised earplugs moulded from a silicone-based putty (President Putty, Coltene Whaledent, Mahwah, NJ, USA) placed in the external auditory meatus, neonatal earmuffs (MiniMuffs, Natus Medical Inc, San Carlos, CA, USA) and an acoustic hood positioned over the shell. All sequences included a 5 second initial slow ramp-up in acoustic noise to avoid eliciting a startle response.

T2-weighted images were acquired using a multi-slice turbo spin echo (TSE) sequence, acquired in two stacks of 2D slices (in sagittal and axial planes), using parameters: TR: 12 s; TE: 156 ms, flip angle: 90°, slice thickness: 1.6 mm acquired with an overlap of 0.8 mm; in-plane resolution: 0.8 × 0.8 mm, scan time: 3:12 min per stack. The T1-weighted volumetric magnetisation prepared rapid acquisition gradient echo (MPRAGE) acquisition parameters were as follows: TR: 11 ms, TE: 4.6 ms, TI:  713 ms, flip angle: 9°, acquired voxel size: 0.8 × 0.8 × 0.8 mm, FOV: 145 × 145 × 108 mm, SENSE factor: 1.2, scan time: 4:35 min. Quantitative flow imaging was performed using velocity sensitised phase contrast imaging, with a single-slice T1-weighted fast field echo sequence. Scan parameters were: field of view (FOV): 100 × 100 mm^2^, acquisition resolution: 0.6 × 0.6 × 4.0 mm^2^, TR: 6.4 ms, TE: 4.3 ms, flip angle: 10°, 20 repetitions, maximal encoding velocity (vENC): 140 cm/s, scan time: 71s.

### Structural image reconstruction

T2-weighted images were reconstructed following the scan using a dedicated motion correction algorithm. Retrospective motion-corrected reconstruction^34,35^ and integration of the information from both acquired orientations^36^ were used to obtain 0.8 mm isotropic T2-weighted volumes with significantly-reduced motion artefacts.

### Brain region and tissue segmentation

Motion-corrected T2-weighted images were segmented into tissue type and 87 brain regions using an automated, validated, neonatal-specific pipeline^37,38^ based on the Expectation–Maximisation (EM) technique^39^, which was optimised for our acquisition parameters. For more details on the individual parts of the segmentation pipeline, we refer the reader to^37,38,40^. Each tissue segmentation was manually inspected for accuracy using ITK-SNAP^41^, and minor corrections performed if necessary.

### Gyrification index calculation

**Figure 3:**
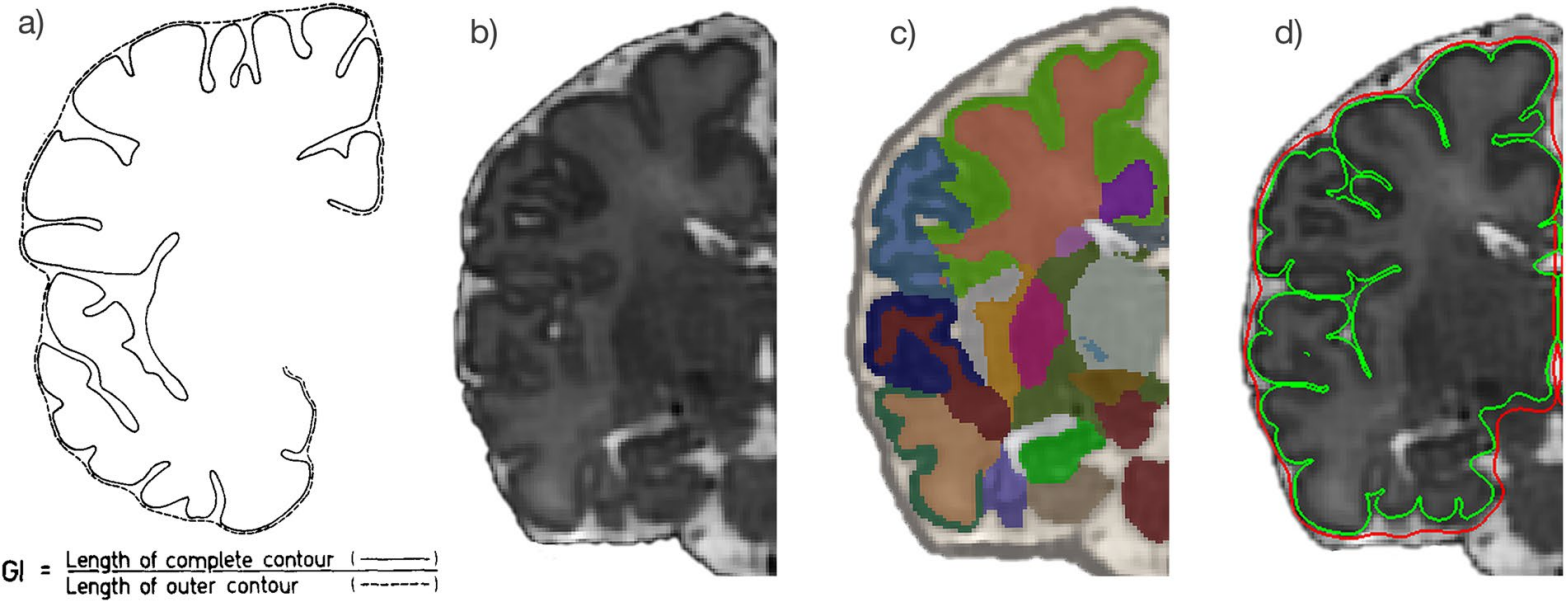
Demonstration of the calculation of gyrification index. (**a**) Original description of gyrification index in histology setting^12^, (**b**) Neonatal brain-extracted T2 volume, (**c**) Automatic segmentation, (**d**) Pial surface mesh (green) and superficial surface (red) created from the segmentation, used to calculate the gyrification index. Figure 3a reproduced from The human pattern of gyrification in the cerebral cortex, Zilles, K., Armstrong, E., Schleicher, A. & Kretschmann, H. J. Anat. Embryol. (Berl). 179, 173–179 (1988). Copyright Springer-Verlag 1988. With permission of Springer.

Gyrification index was defined as the ratio of the cortical pial surface area and the surface area of the superficial surface enclosing the pial surface^12,13,42^. This ratio was calculated for each subject using pial surfaces constructed from the combined grey/white matter mask derived from the segmentations, using a previously-published method^43^, as demonstrated in Fig. 3. Final cortical surfaces were cleaned using median filtering and Laplacian smoothing. The superficial surface was reconstructed using marching cubes from the morphologically closed combined grey/white matter mask. Morphological closing (performed by 3 dilations followed by 2 erosions) removed small sulci and generated a mask that enclosed the original cGM/WM mask. The gyrification index was calculated initially for the whole brain, and then separately for each major brain region (frontal, parietal, temporal, occipital), using appropriate combinations of the 87 segmented brain regions^40^.

### Cerebral blood flow and cerebral oxygen delivery

**Figure 4:**
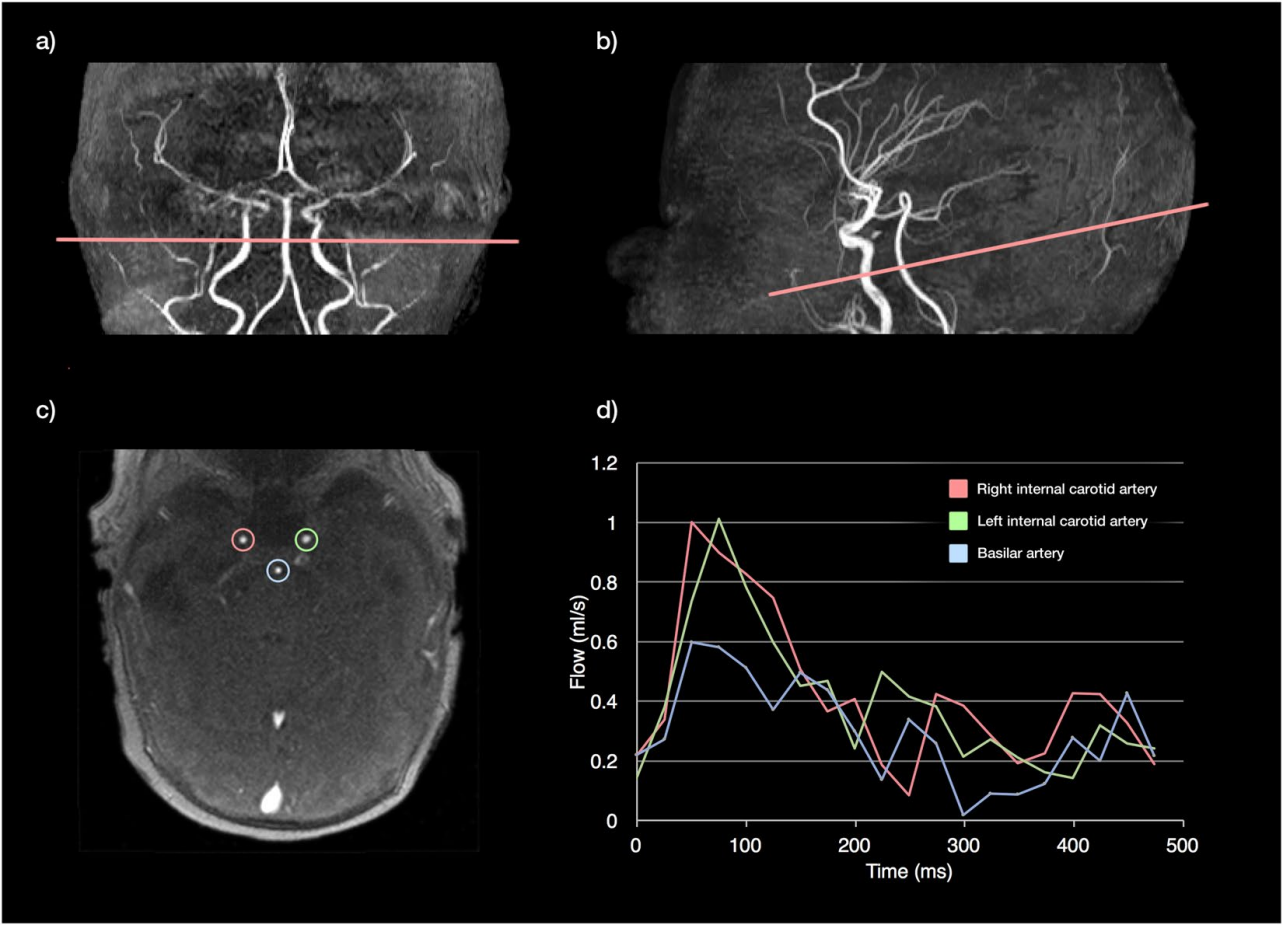
Phase contrast measurements of the cerebral vessels in the neonatal brain. The plane is planned from a 3D non-contrast angiogram in both coronal (**a**) and sagittal planes (**b**), aiming for the C4 segment of the internal cerebral arteries (ICA) where all three vessels are running approximately parallel). Following the scan, regions of interest are drawn around the three major cerebral vessels: left (green) and right (red) ICAs, and basilar artery (blue), and these regions are propagated through the cardiac cycle. Flow curves can then be derived across the cardiac cycle (**d**).

To calculate cerebral blood flow, we used a previously-published scanning protocol, acquired in a plane perpendicular to both internal carotid and basilar arteries, at the level of the sphenoid bone^44^, as demonstrated in Fig. 4.

Regions of interest were drawn manually around the three vessels, using Segment v2.0 R4800^45^, and flow curves generated (Fig. 4.). An estimate of total cerebral blood flow (CBF) was calculated from the sum of these vessels. This disregards blood flow to some areas of the cerebellum, which is provided by branches of the vertebral arteries and constitutes less than 3% of the total flow to the brain in healthy adults^46^.

Haemoglobin (Hb) levels were measured as part of routine clinical care in all patients at a median of 4 days (range 0–10 days) prior to the scan. All newborns with more infrequent Hb monitoring were clinically stable, and we believe all Hb measurements used were representative of levels at time of scan. Arterial oxygen saturation (SaO_2_) was measured at the time of scan using a Masimo Radical-7 monitor (Masimo Corp, Irvine, CA) applied to the right hand.

Cerebral oxygen delivery (CDO_2_) was calculated using the following formulae^47^:

$$CD{O}_{2}({\rm{mlO}}2/{\rm{\min }})=Sa{O}_{2}\times [{\rm{Hb}}]\,(g/\mathrm{dL})\times 1.36\times [{\rm{CBF}}](\mathrm{ml}/{\rm{\min }})$$

where 1.36 is the amount of oxygen bound per gram of haemoglobin at 1 atmosphere (Hüfner’s constant)^18^.

### Regional cerebral oxygen saturation (rScO_2_)

All newborns had near-infrared spectroscopy rScO_2_ estimations performed immediately after the scan using a FORE-SIGHT Elite cerebral oxygenation monitor (Casmed, Branford, CT, USA) with neonatal sensor, which was placed over the left frontal region. Measurements were taken for at least three minutes with a sampling frequency of 2 seconds. The output was analysed to obtain mean and standard deviation using a script written in Python (Python Software Foundation, https://www.python.org/). To assess repeatability, the measurement was repeated in a subset of subjects, by disconnecting and removing the sensor from the baby, and reapplying it in a similar location.

### Statistical analysis

We automatically matched healthy newborns with babies born with congenital heart disease using an automated method that minimises overall group Euclidean distances between GA and PMA, written in Matlab (R2016, The MathWorks, Inc., Natick, MA, US). We compared group characteristics in newborns with CHD to the healthy control group with the Mann–Whitney U test for continuous data, and Fisher’s exact test for categorical variables. Analysis of Covariance (ANCOVA) tests were performed to assess volumetric measurements and gyrification index differences between groups; PMA at scan was included as a covariate. For subsequent regional analyses, outside of the primary hypothesis test, multiple comparisons correction was not performed. Agreement between consecutive readings of rScO2 was analysed using Pearson correlation. Regression analysis was used to compare CDO_2_ to brain parameters. Z-scores for birth weight and head circumference were calculated using the SITAR R package^48^, using UK-WHO 2006 population reference data^49,50^. Statistical analysis was performed with SPSS Statistics v24 (IBM) and graphs were prepared using R Studio (v1.0.136, RStudio Inc, Boston MA). Three-dimensional visualisations were performed in ParaView^51^.

### Data availability

The datasets generated during and/or analysed during the current study are available from the corresponding author on reasonable request.

## Electronic supplementary material

Supplementary Information
