# Supplementary material for: Impaired development of the cerebral cortex in infants with congenital heart disease is correlated to reduced cerebral oxygen delivery
Source: Sci Rep. 2017 Nov 8;7:15088. doi: 10.1038/s41598-017-14939-z (PMC5678433; doi:10.1038/s41598-017-14939-z)
Supplement: Supplementary file 1 — Supplementary Information [file 41598_2017_14939_MOESM1_ESM.pdf]

Supplementary Information

Impaired development of the cerebral cortex in infants with congenital heart disease is correlated to reduced cerebral oxygen delivery

Christopher J. Kelly, Antonios Makropoulos, Lucilio Cordero-Grande, Jana Hutter, Anthony Price, Emer Hughes, Maria Murgasova, Rui Pedro A G Teixeira, Johannes K. Steinweg, Sagar Kulkarni, Loay Rahman, Hui Zhang, Daniel C Alexander, Kuberan Pushparajah, Daniel Rueckert, Joseph V. Hajnal, John Simpson, A. David Edwards, Mary A. Rutherford, Serena J. Counsell

Contribution of cerebral blood flow and arterial saturations to CDO2

Cerebral blood flow (CBF) correlated more strongly ($R^2 = 0.643$, $p < 0.001$) with CDO2 than arterial saturations ($R^2 = 0.107$, $p = 0.119$), suggesting that CBF is more responsible for the reduction in CDO2.

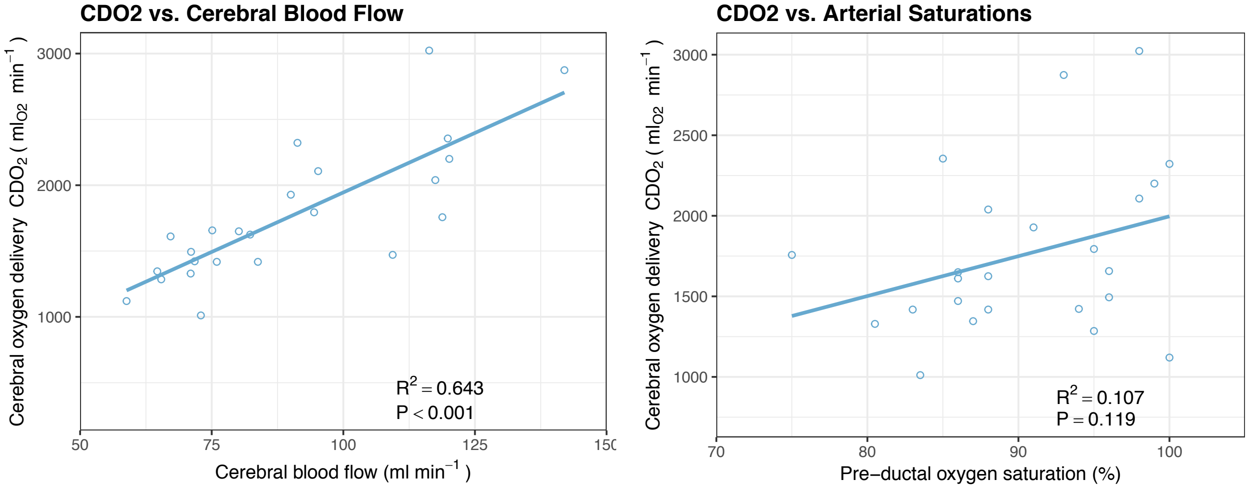

Supplementary Figure S1 Left: relationship between CDO2 and CBF. Right: relationship between CDO2 and preductal arterial oxygen saturations.

Difference in cerebral haemodynamic variables between different CHD groups

There was a trend towards higher cerebral blood flow and cerebral oxygen delivery in right-sided lesions, and lower flow in left-sided and abnormal mixing groups (Supplementary Figure S2). However, at the sub-group level, small samples sizes make it difficult to draw strong conclusions.

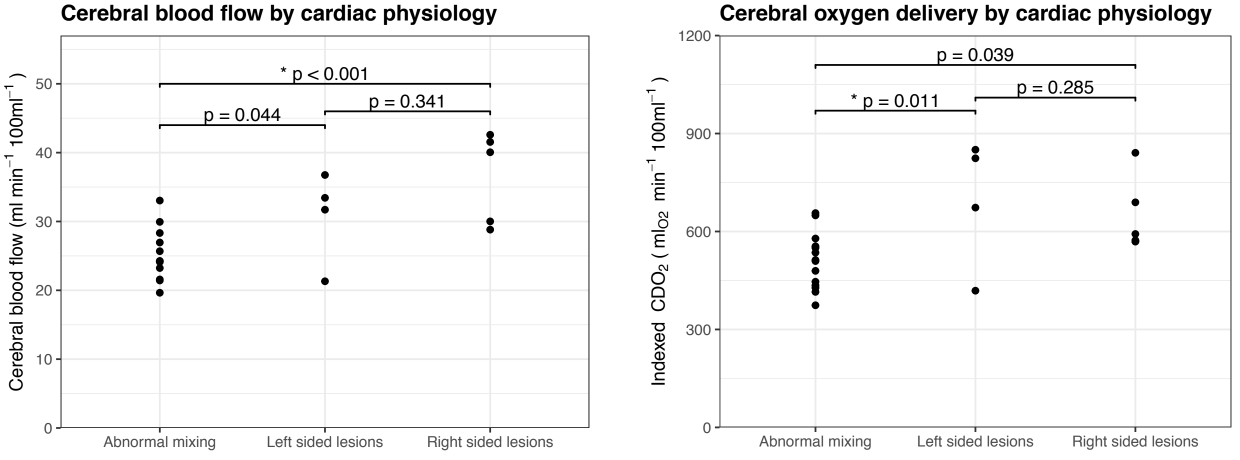

Supplementary Figure S2 Left: Cerebral blood flow by cardiac physiology. CHD with abnormal mixing appears to result in lower flow vs right-sided lesions, although sample sizes are small. Right: Cerebral oxygen delivery (expressed per 100ml brain volume) by cardiac physiology. Statistics performed using general linear model with gestational age at scan as a covariate. Bonferroni correction leads to a significance threshold of 0.017.
